# Supplementary material for: Functional and multiscale 3D structural investigation of brain tissue through correlative in vivo physiology, synchrotron microtomography and volume electron microscopy
Source: Nat Commun. 2022 May 25;13:2923. doi: 10.1038/s41467-022-30199-6 (PMC9132960; doi:10.1038/s41467-022-30199-6)
Supplement: Supplementary file 1 — Supplementary Information [file 41467_2022_30199_MOESM1_ESM.pdf]

Functional and multiscale 3D structural investigation of brain tissue through correlative *in vivo* physiology, synchrotron micro-tomography and volume electron microscopy

Carles Bosch[1 *], Tobias Ackels[1,2], Alexandra Pacureanu[1,2,3], Yuxin Zhang[1,2], Christopher J Peddie[4], Manuel Berning[5,6], Norman Rzepka[6], Marie-Christine Zdora[7,8,9], Isabell Whiteley[1,2], Malte Storm[8,10], Anne Bonnin[11], Christoph Rau[8], Troy Margrie[12], Lucy Collinson[4], and Andreas T Schaefer[1,2 *]

1 Sensory Circuits and Neurotechnology Lab., The Francis Crick Institute, London, UK
2 Department of Neuroscience, Physiology and Pharmacology, University College London, UK
3 ESRF, The European Synchrotron, Grenoble, France
4 Electron Microscopy STP, The Francis Crick Institute, London, UK
5 Department of Connectomics, Max Planck Institute for Brain Research, Frankfurt am Main, Germany
6 scalable minds GmbH, Potsdam, Germany
7 Department of Physics and Astronomy, University College London, London, UK
8 Diamond Light Source, Harwell Science and Innovation Campus, Didcot, UK
9 School of Physics and Astronomy, University of Southampton, Highfield Campus, Southampton, UK
10 Institute of Materials Physics, Helmholtz-Zentrum Hereon, Geesthacht, Germany
11 Paul Scherrer Institut, Villigen, Switzerland
12 Sainsbury Wellcome Centre, University College London, London, UK
* Corresponding authors

Supplementary Figures

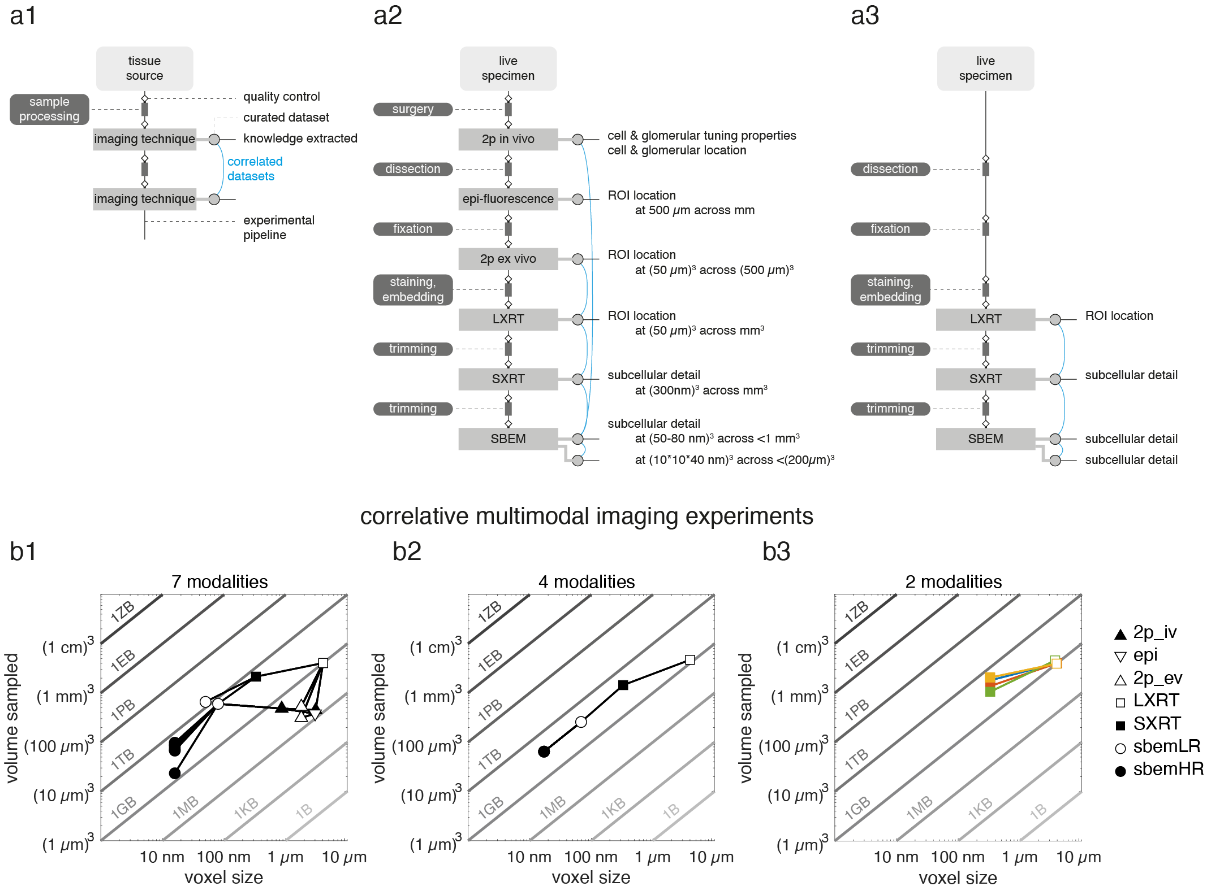

Supp. Fig. 1

Comparison of correlative multimodal imaging (CMI) pipelines.

(a) Flowchart diagram **(a1)**, applied to a CMI pipeline including 6 imaging techniques **(a2)** and to a CMI pipeline including 3 imaging techniques **(a3)**. This diagram shows the elements that might affect the throughput of a CMI pipeline and is helpful for identifying bottlenecks. Samples are processed before every imaging technique to match compatibility requirements and enhance signal detection. A quality control step is present before any sample preparation and imaging step, aimed to maximise the success rate of the following steps. Raw data has to be processed to generate a curated dataset. Curated datasets can be correlated, thereby enabling to link the information obtained in their respective analyses and augmenting the knowledge extracted from the pipeline.

(b) Correlated datasets in a CMI experiment including 7 modalities **(b1),** 4 modalities **(b2)**, and in four experiments including the same 2 modalities each **(b3)**. Note that some imaging techniques can image the same specimen at different sampling rates, providing more than one modality (e.g. SBEM high vs low-resolution). Furthermore, some techniques allow imaging multiple regions of interest, thereby providing more than one dataset per modality (e.g. SBEM high-resolution datasets in **b1**). For each dataset, the x axis shows the "voxel size" being the cubic root of the product of the acquired voxel size in x,y,z - thereby representing the length of the side of a voxel if voxels were isotropic. The y axis shows total volume sampled. In this diagram, datasets containing the same number of voxels are distributed along a diagonal. For reference, diagonals hosting datasets of each order of magnitude are shown in shaded greys and their sizes are indicated assuming they are uncompressed 8-bit images. The dataset marker represents the imaging modality: *2p_iv*, 2-photon *in vivo* Ca^{2+}; *epi*, epifluorescence of the dissected slab; *2p_ev*, 2-photon *ex vivo* imaging of the fixed slab; *LXRT*, laboratory X-ray µCTs; *SXRT*, synchrotron X-ray computed tomography with propagation-based phase contrast; *sbemLR* and *sbemHR*, serial block-face electron microscopy at low and high resolution

respectively. Datasets spatially correlated are linked with an edge. All plots represent CMI pipelines reported in this study. **(b1)** and **(b2)** contain one single experiment each. In **(b3)**, datasets belonging to each experiment are shown in the same colour.

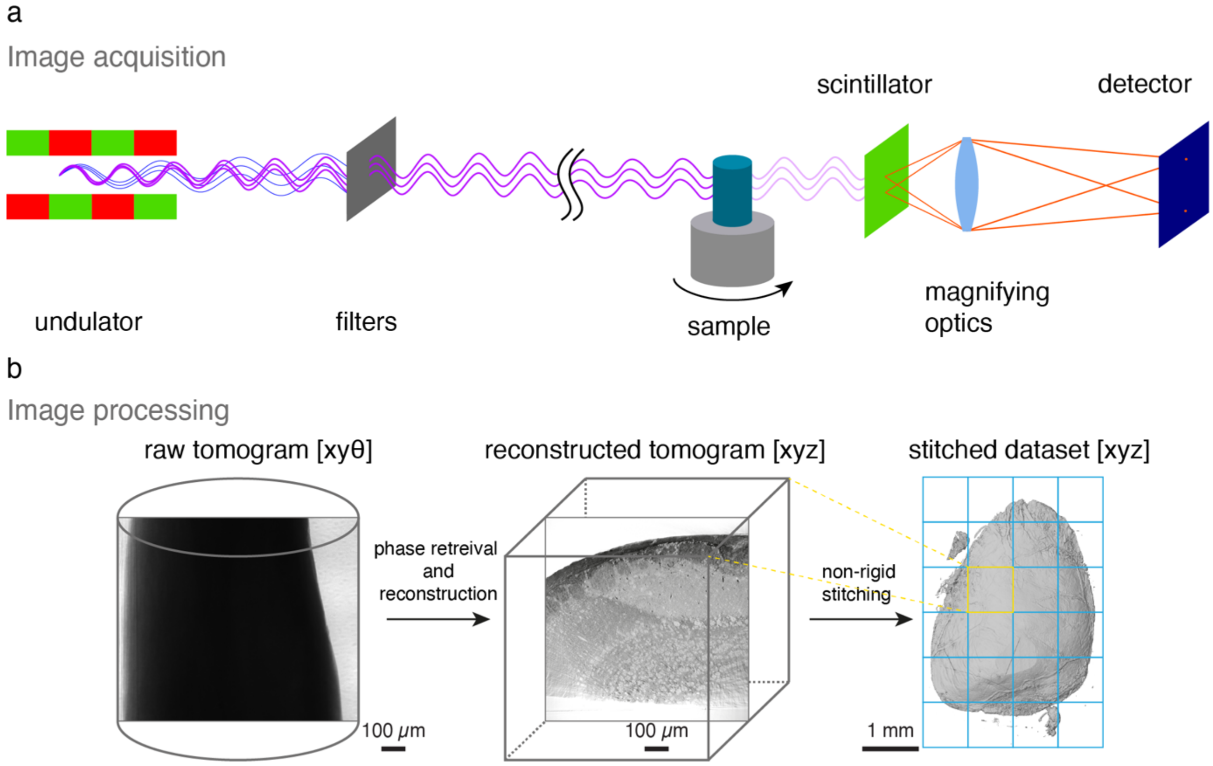

Supp. Fig. 2

Data acquisition and processing in a synchrotron X-ray CT experiment.

(a) Simplified schematic of the SXRT experiment at Diamond I13-2. The undulator (alternating magnetic structures, depicted as red/green) creates polychromatic X-rays from the stored electron beam. X-ray filters remove the lower energy UV and X-ray radiation and only leave the tailored X-ray spectrum for the experiment.

The experimental station itself is situated about 230 m downstream of the undulator, and the sample is mounted on a rotation stage. After interacting with the sample via absorption and scattering, the X-rays reach a scintillator crystal which converts them to visible light. Magnifying optics project an image of the scintillator on the chip of the sCMOS camera. The images are then further processed and reconstructed.

(b) Simplified schematic of the image processing required to generate datasets from the synchrotron X-ray CT experiment. The sample is rotated and a projection of the sample on the detector is recorded at each angular step θ, thereby generating a x,y,θ dataset. This is subsequently reconstructed into a x,y,z dataset. The sample is moved so that overlapping 3D tiles are captured covering the entire region of interest of the sample. The reconstructed x,y,z tiles are stitched together using a non-rigid algorithm to ensure a smooth transition at the boundaries. The stitched dataset represents an x,y,z volume covering the entire region of interest.

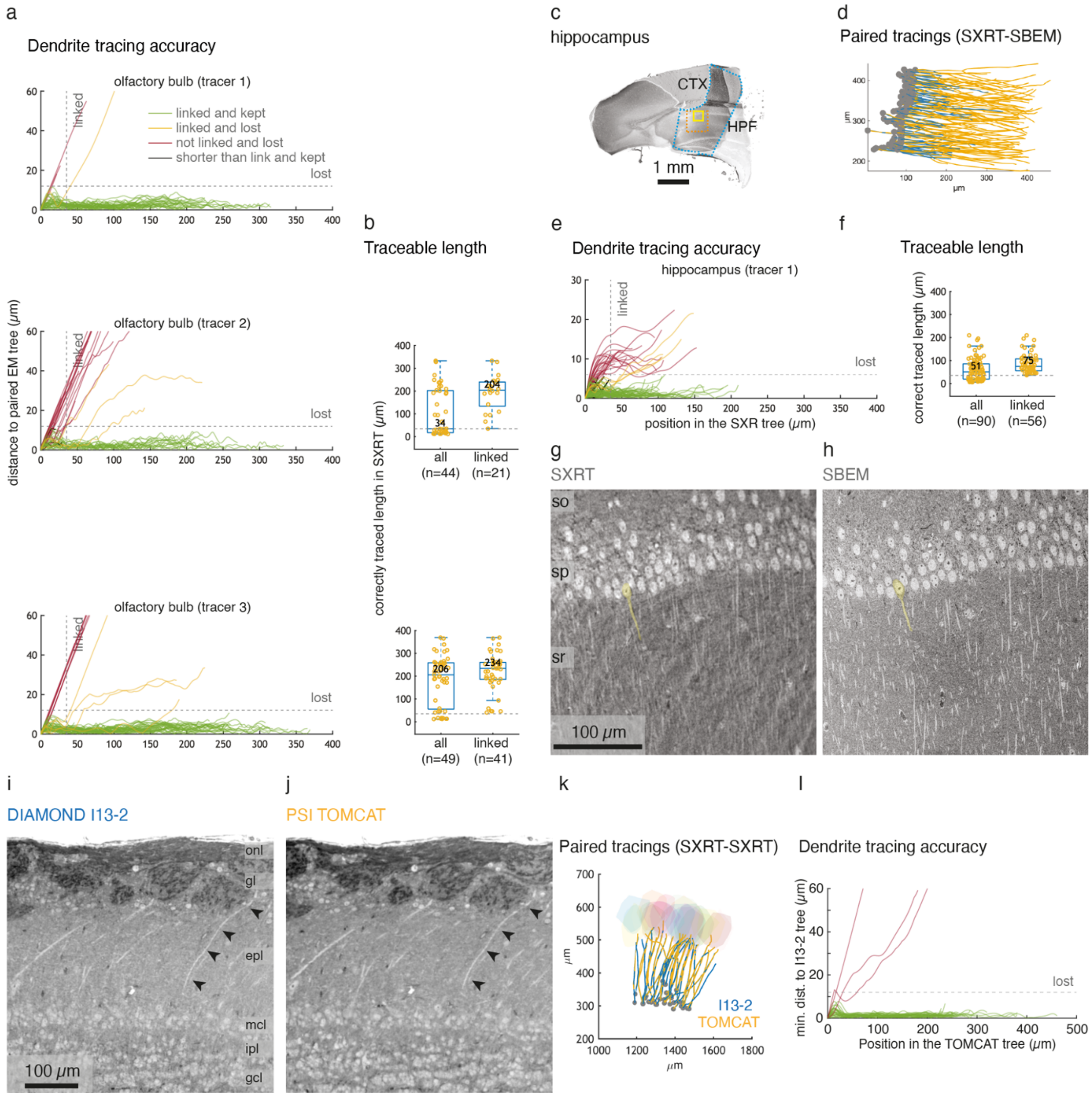

Supp. Fig. 3

Apical dendrites of multiple brain regions are traceable in SXRT datasets

(a) Distance between SXRT tracing and EM ground truth tracing of the same OB mitral cell apical dendrite for cells analysed in **Fig. 2**. For each point on a SXRT dendrite tracing, the minimum distance to its paired EM tracing is plotted. (Prior to distance calculation SXRT and SBEM tracings were warped into the same dataset space, see methods). Each SXRT-SBEM pair of traces (i.e. one cell's apical dendrite) is represented by a line, and is classified into four groups defined by two thresholds: a SXRT tracing is considered as "lost" if the average distance to its paired EM tree exceeds 12µm, otherwise as "kept"; a SXRT tracing is considered as "linked" if it is not lost before 35µm from the start of the tracing, otherwise as "not linked". Lines belonging to each group are color-coded accordingly (green, yellow, red and black lines). Dashed lines indicate thresholds 'linkage' (35 µm) and 'lost' (12 µm). Three independent tracers performed the same task, and their individual accuracies are plotted separately.

(b) Traceable length of all apical dendrites and of the correctly linked ones in SXRT for tracers #2 and #3, as shown in **Fig. 2e** for tracer #1. All tracers traced the same set of dendrites from the same dataset. The box covers the 25% to 75% percentile range; the middle bar represents

the median value (printed above); the whiskers extend to the most extreme data points that are not an outlier (defined as outside of the 1.5x interquartile range). The grey dashed line marks the lost threshold used.

(c) Volume of a mouse hippocampus obtained with LXRT. Region acquired with SXRT is indicated with a blue dashed line. The region acquired with low-resolution SBEM (80x80x50 nm^3 voxels) is indicated with an orange dashed line (same datasets as in **Fig. 4a)**. Below, synchrotron X-ray CT **(g)** and SBEM **(h)** detail of the highlighted region in yellow.

(d) Paired apical dendrites traced in SXRT (blue lines) and SBEM (orange lines).

(e) Same as **(a)** for CA1 pyramidal neurons. Dashed lines indicate thresholds 'linkage' (35 µm) and 'lost' (6 µm).

(f) Same as **(b)** for CA1 pyramidal neurons. Boxes are plotted as described in **(b)**.

(i-j) Same region imaged with SXRT at the Diamond beamline I13-2 **(i)** and at the SLS beamline TOMCAT **(j)**.

(k) Paired apical dendrites of the same mitral cells traced in both SXRT datasets (blue lines: I13-2, orange lines: TOMCAT). Overlap between paired traces indicates a good agreement between the same dendrites traced in the two datasets. Contours of glomeruli are displayed as coloured blobs.

(l) Distance between traces of paired dendrites. 91% of the dendrites were accurately traced in both datasets (30 out of 33 dendrites traced in both datasets).

onl, olfactory nerve layer; *gl*, glomerular layer; *epl*, external plexiform layer; *mcl*, mitral cell layer; *ipl*, inner plexiform layer; *gcl*, granule cell layer; *CTX*, cerebral cortex; *HPF*, hippocampal formation; *so*, stratum oriens; *sp*, stratum pyramidale; *sr*, stratum radiatum.

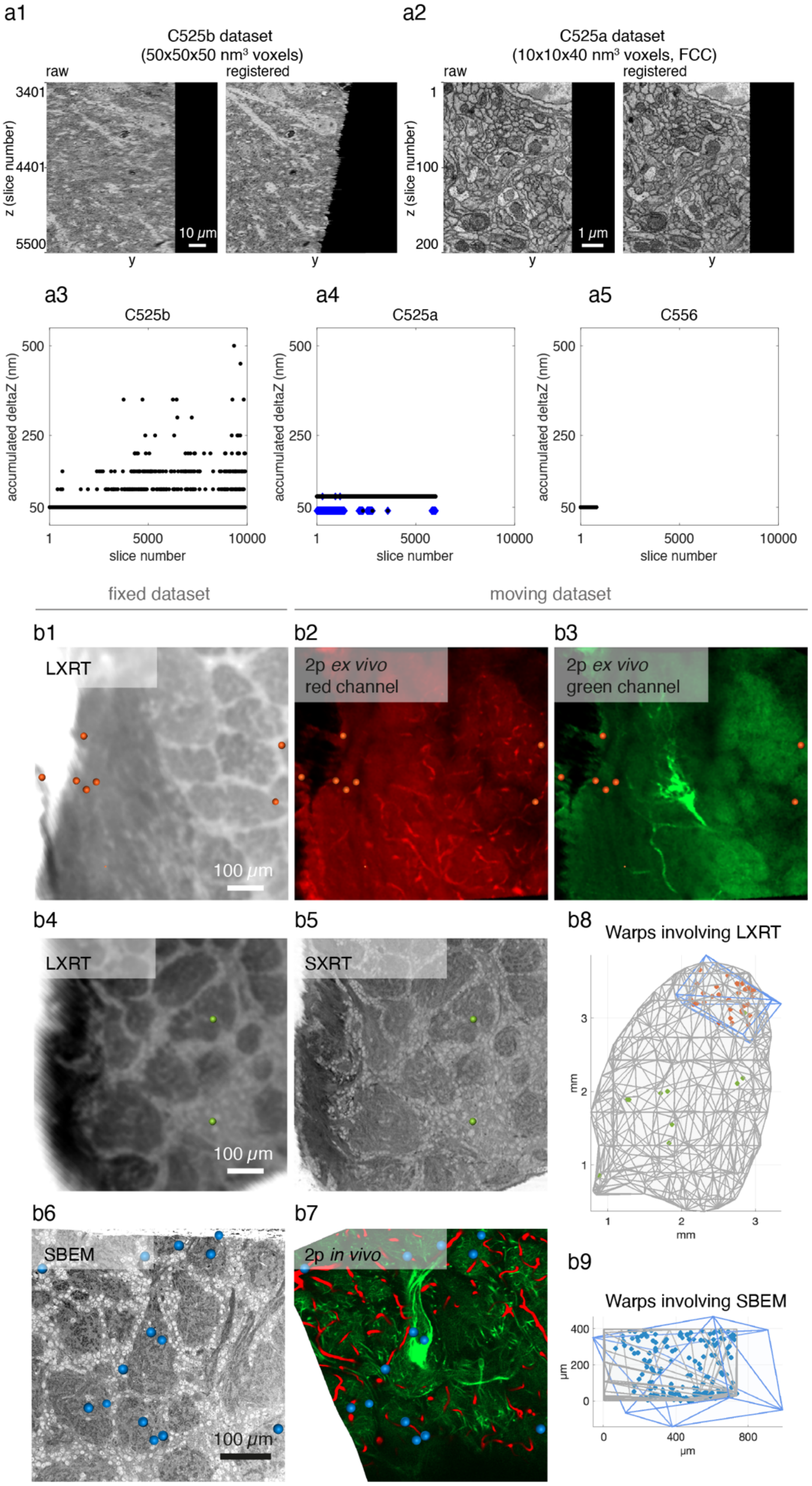
a1
C525b dataset
(50x50x50 nm³ voxels)
raw
registered
3401
4401
5500
z (slice number)
10 μm
y
a2
C525a dataset
(10x10x40 nm³ voxels, FCC)
raw
registered
1
100
200
1 μm
a3
C525b
a4
C525a
a5
C556
accumulated deltaZ (nm)
500
250
50
1
5000
10000
slice number
fixed dataset
moving dataset
b1
LXRT
100 μm
b2
2p ex vivo
red channel
b3
2p ex vivo
green channel
b4
LXRT
b5
SXRT
b8
Warps involving LXRT
mm
b6
SBEM
b7
2p in vivo
b9
Warps involving SBEM
μm
400
200
0
800

Supp. Fig. 4
Image registration for serial block-face electron microscopy datasets, and warping datasets from different modalities using manually seeded landmarks.
(a) Registered outcomes from three experiments involving SBEM. **(a1)** Unregistered (left) and registered (right) yz reslice of the same region in the C525b dataset. **(a2)** Unregistered (left) and registered (right) yz reslice of the same region in the C556 dataset. **(a3-a5)** Summary plots displaying the z distance between every two consecutive slices in the curated dataset, for datasets in C525b **(a3)**, C525a **(a4)** and C556 **(a5)**. In C525a **(a4)**, the experiment consisted of several high-resolution datasets spaced 40 nm (blue diamonds) in z, while a low-resolution dataset at 80 nm spacing in z covered the entire volume (black dots). In C556 **(a5)**, both high- and low-resolution datasets covered the same region in z, had the same distance between slices, and no slices were discarded for any - therefore are all shown as black dots.
(b) Warping datasets of different modalities to a common space using manually seeded landmarks. Details of the same region in both modalities and a group of local landmarks in context are shown for 2-photon *ex vivo* microscopy datasets warped into LXRT **(b1-3**, orange landmarks), LXRT into SXRT **(b4-b5**, green landmarks) and *in vivo* 2-photon anatomical dataset onto SBEM **(b6-b7**, blue landmarks). The landmarks are shown in **(b8-9)** in the context of the datasets involved, with the field of view of the 2-photon datasets outlined in blue and the tissue boundaries in X-ray or EM datasets outlined in grey.

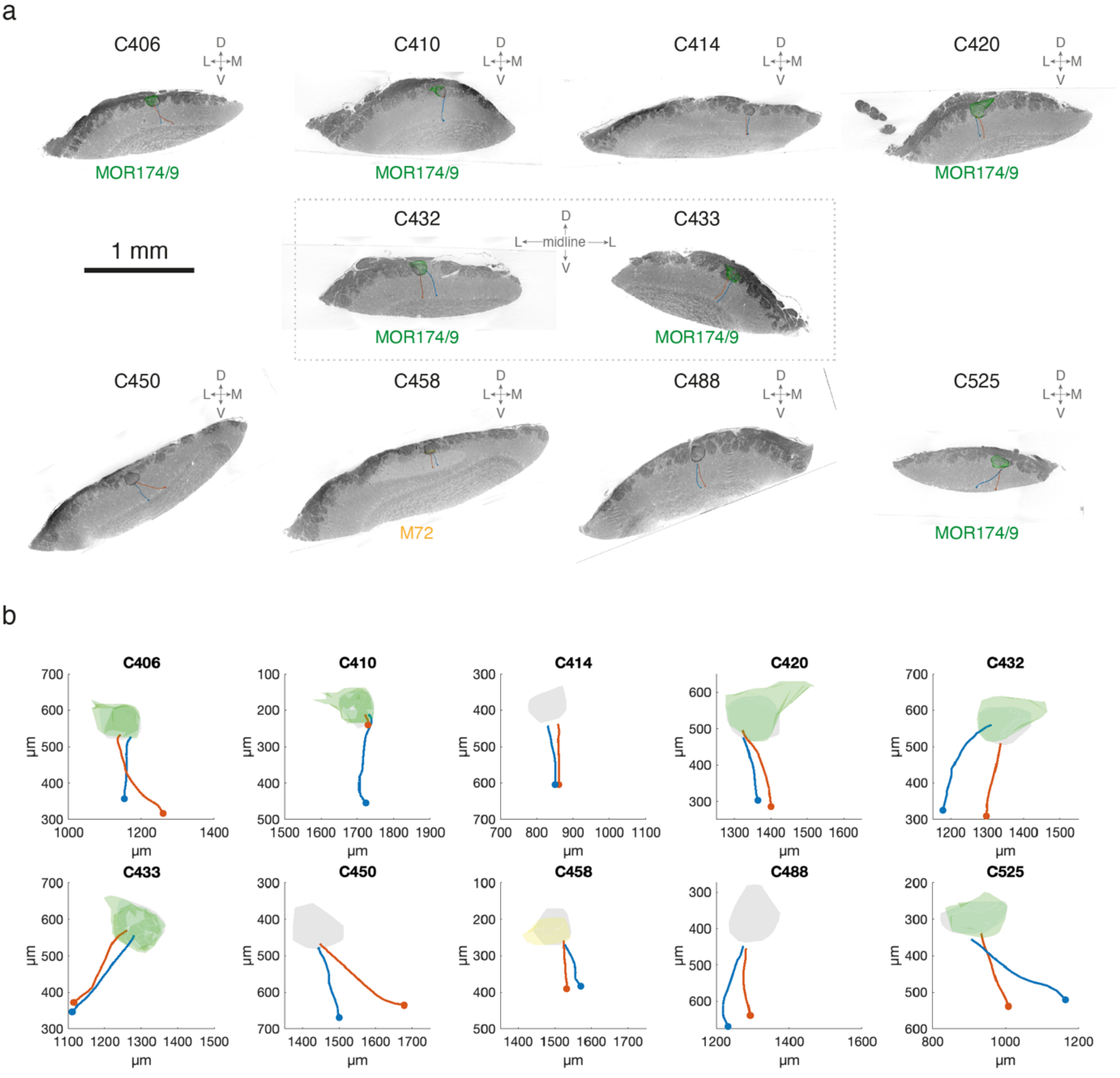

Supp. Fig. 5

Sister mitral cells from genetically-targeted glomeruli.

(a) SXRT virtual coronal cross-sections of 10 olfactory bulb samples from 9 mice (in one case, both brain hemispheres were sampled). In all cases the genetically-identified glomerulus (MOR174/9 or M72) could be identified through a correlative *ex vivo* 2p, LXRT and SXRT approach. Two sister mitral cells (blue, orange) were traced on a glomerulus in each dataset, and a grey mesh indicates the contour of that glomerulus traced in the SXRT dataset. In those cases where the glomerulus shown corresponds to the genetically-targeted one, coloured meshes display the glomerular contour of the fluorescently-labelled glomerulus, traced on the *ex vivo* 2p dataset and warped onto the SXRT space (green for MOR147/9, yellow for M72).
(b) Close-up view of the same annotations, more clearly displaying the cell bodies, apical dendrites and glomeruli.
D, dorsal; *V*, ventral; *L*, lateral; *M*, medial.

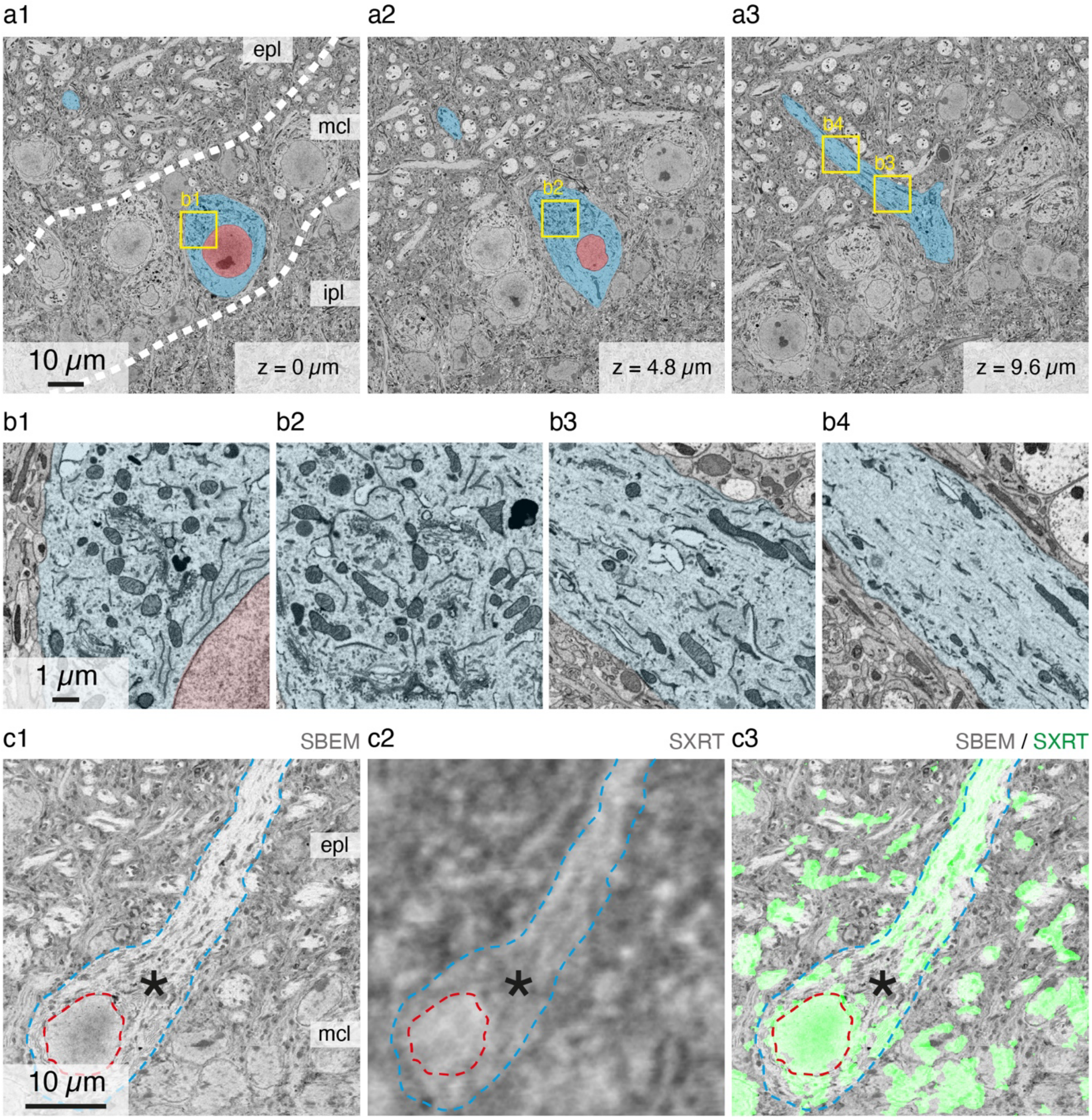

Supp. Fig. 6
Initial segment of the apical dendrite of a mitral cell.
(a) Mitral cell (nucleus in red, cytoplasm in blue) shown at three z depths **(a1-3)**. Details of the cytoplasm are shown for four regions: in soma, proximal **(b1)** and distal to nucleus **(b2)**, beginning of apical dendrite **(b3)** and apical dendrite **(b4)**. Note the abundance of electron-dense features in **(b1-3)** - including mitochondria, rough and smooth endoplasmic reticulum, and Golgi apparatus - and how they become much less prominent in **(b4)**.
(c) Correlative SBEM **(c1)** and SXRT **(c2)** of the same region containing a mitral cell. Both datasets were warped to a common space. Nucleus (red dashed line) and cell boundaries (blue dashed line) are labelled, the latter delineating the continuum cell body – apical dendrite. The overlay of both modalities is shown in **(c3)**, with the synchrotron X-ray image thresholded so only data from poorly absorbing regions (e.g. dendrites) is displayed (green channel). Note that in the SXRT dataset both the nucleus and the apical dendrite are well defined whereas the initial segment of the apical dendrite can get blended into the surrounding neuropil (asterisk).
epl, external plexiform layer; *mcl*, mitral cell layer; *ipl*, inner plexiform layer.
The dataset from which the micrographs in **(a, b)** are taken was previously reported in reference [106].

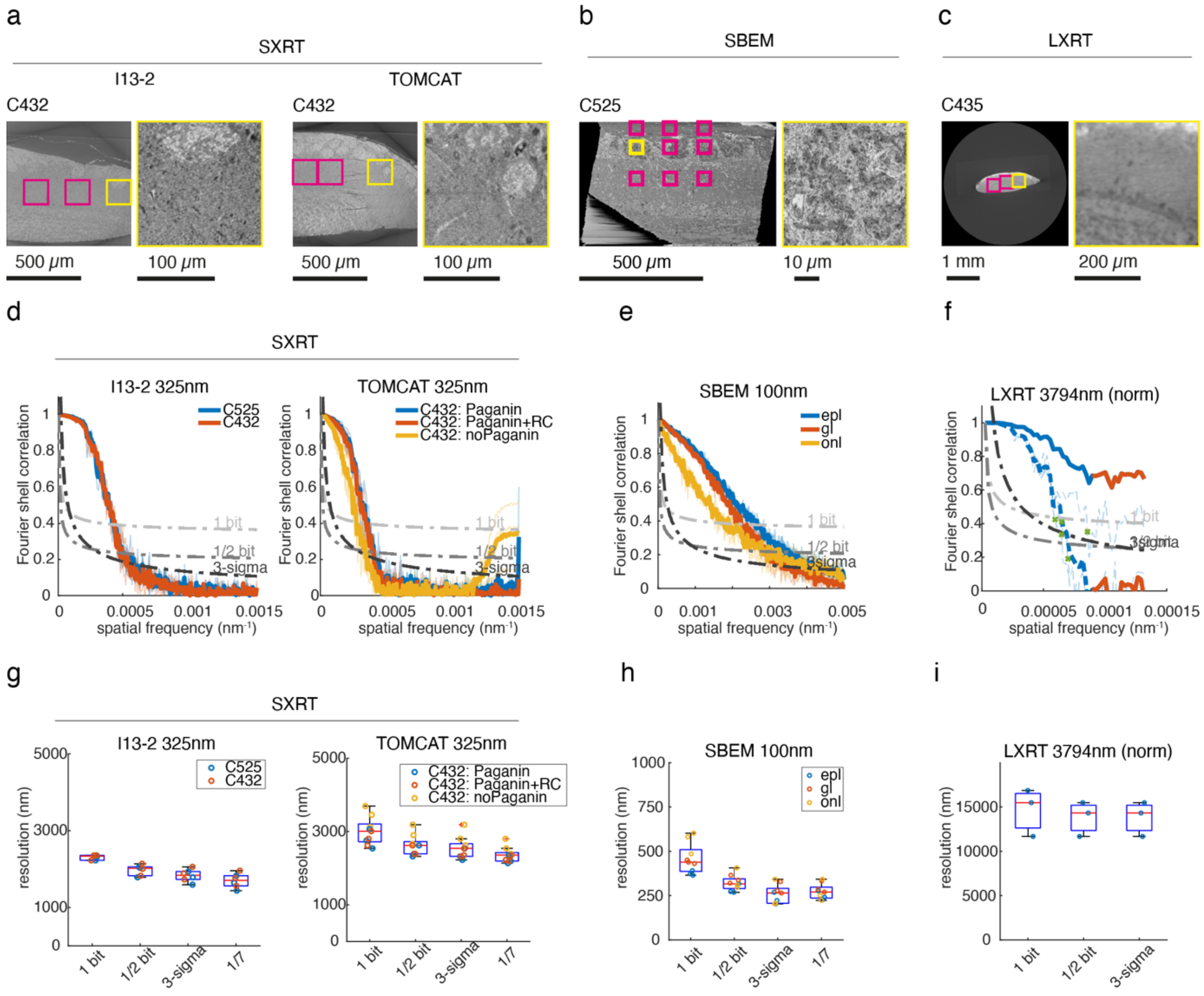

Supp. Fig. 7

Resolution of LXRT, SXRT and SBEM datasets.

Resolution of the datasets obtained across all structural modalities. **(a-c)** Volume ROIs large enough so relevant features would be contained were sampled from the original datasets (500 voxels wide for all cases except for LXRT, where ROIs were 100 voxels wide). For each trace, two volume ROIs acquired independently and containing data of the same volume were compared through FSC analysis against each other. In SXRT **(a)** and LXRT datasets **(c)** half-tomogram reconstructions were generated from odd and even projection subsets. In the SBEM dataset (originally acquired at 50 nm voxel size) **(b)** odd and even slices laterally downscaled by ½ generated equivalent ROIs at 100nm isotropic voxel size. **(d-f)** Fourier shell correlation curves observed in the different imaging modalities. Variability was assessed across specimens, reconstruction algorithms and histological regions. For LXRT **(f)**, the reconstruction algorithm of the half-tomograms was not fully editable and possibly contained some common filtering step, which rendered artefactual high correlations at even 1/(2*pixel) frequency. The average FSC curve (bold solid line) was normalized (bold dashed line) assuming the correlations at frequencies [1/(3*pixel) 1/(2*pixel)] (orange segment) would be null. The same normalization was applied to all individual traces (thin dashed lines) from which resolution measurements were extracted later on (green crosses). **(g-i)** Resolution readouts were obtained for each FSC curve at the point it crossed the resolution criteria for the different imaging modalities. Four commonly used resolution criteria were applied to most cases: 1 bit, ½ bit, 3 sigma and 1/7. *n* = 2 biologically independent samples in **g** (left panel) and *n* = 1 in **g** (right panel), **h**, **i**. *n'* = 3 regions of interest originating from the same dataset in **g**, *n'*=9 in **h** and *n'*=3 in **i**. The boxes in the box plots cover the 25% to 75% percentile range; the middle

bar represents the median value; the whiskers extend to the most extreme data points that are not an outlier (defined as outside of the 1.5x interquartile range).

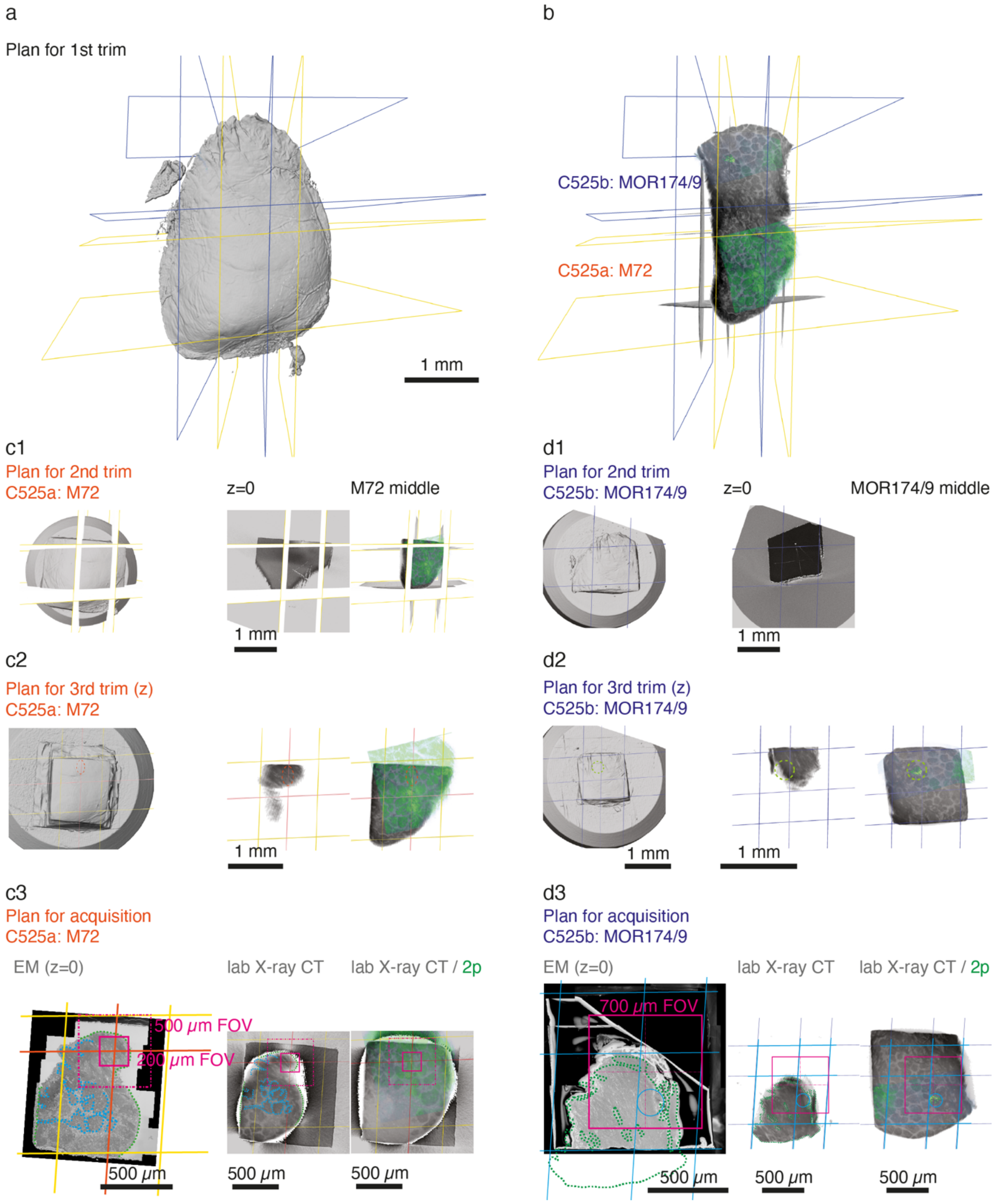

Supp. Fig. 8

Specimen trimming into a geometry compatible with SBEM.

(a) Volume reconstruction of the LXRT dataset of the stained slab. A virtual section is shown in **(b)**, overlaid with warped 2-photon datasets taken of the fixed tissue, which carry information on the location of two genetically-tagged glomeruli, M72 and MOR174/9. Regions that contained both circuits were virtually resliced with yellow and blue planes, respectively. These images were then used to guide the mechanical trimming of the specimen, which was again scanned with LXRT, the dataset warped into the common framework, and iteratively trimmed until the sample was <1 mm^3 in size for both M72 **(c1-2)** and MOR174/9 **(d1-2)**

specimens. The block-face was then carefully shaved off **(c2, d2)** rendering a specimen with enough exposed tissue in the block-face to accurately predict the location of the ROI underneath **(c3, d3)**. This last block-face enabled configuring the field of view of the datasets to be acquired with SBEM.

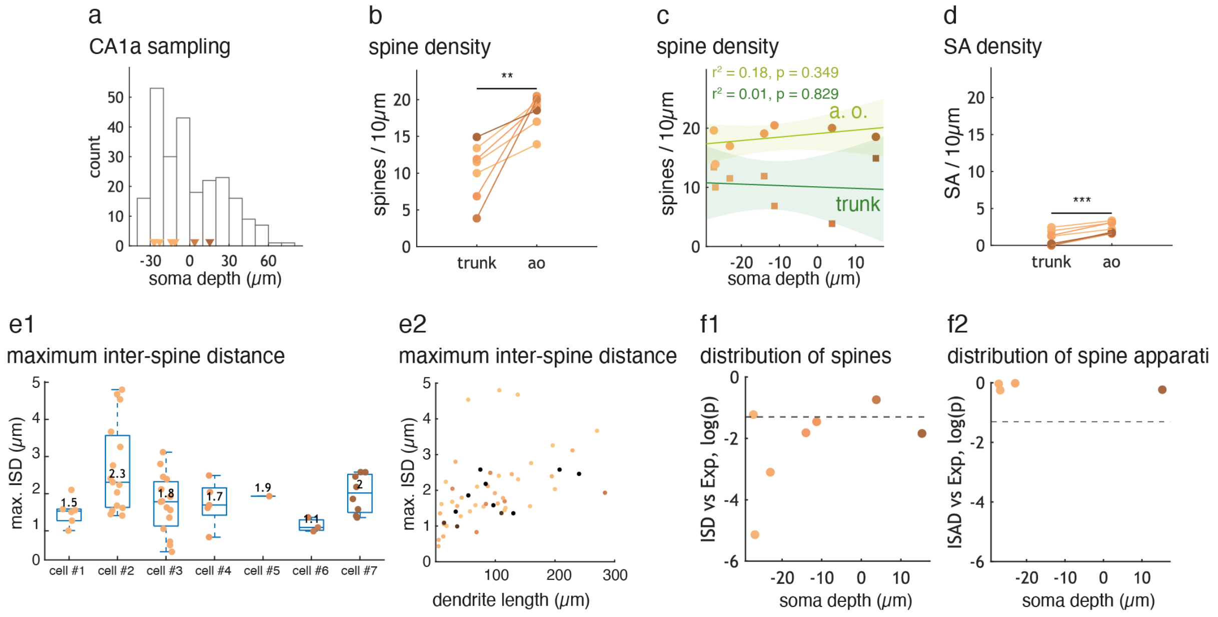

Supp. Fig. 9

Density and distribution of spines in the *stratum radiatum* dendrites of CA1a neurons.

(a) Histogram of the soma depths of all CA1a somata analysed with SXRT in **Fig. 4**, alongside arrowheads pointing to the positions of the seven neurons with follow-up correlative SBEM. Colour coding according to soma depth, as in **Fig. 4b**.

(b) Average spine density in trunk versus apical oblique dendrites ($n = 7$ cells from one dataset, two-tailed paired t-test, $p = 0.0049$), **(c)** plotted as a function of soma depth ($n = 7$ cells, linear regression, $p = 0.349$, 0.829 for apical oblique and trunk dendrites, respectively). The shaded area represents the 95% confidence interval of the linear regression line.

(d) Average spine apparatus density in trunk versus apical oblique dendrites ($n = 7$ cells, two-tailed paired t-test, $p = 0.000081$).

(e) Maximal inter-spine distance, grouped per cell **(e1)** and as a function of the length of each dendritic branchlet analysed **(e2)**. $n = 6, 15, 15, 5, 1, 3, 8$ dendritic branchlets for cells #1-7, respectively. For each cell, the median is displayed in the box plot. Each dendritic branchlet is represented by a dot, dots coloured according to the soma depth of the cell. The box covers the 25% to 75% percentile range; the middle bar represents the median value; the whiskers extend to the most extreme data points that are not an outlier (defined as outside of the 1.5x interquartile range). Note there is an upper bound on inter-spine distances, with no spines found further apart than 2 μm in most dendrites of all cells analysed. Large inter-spine distances are therefore less frequent than expected by chance.

(f) Probability of spines **(f1)** and spine apparati **(f2)** being randomly distributed in apical oblique dendrites (comparing their inter-spine and inter-spine apparatus distances to a Poisson distribution of spines).

(* $p<0.05$, ** $p<0.005$, *** $p<0.0005$, ns non-significant in all panels).

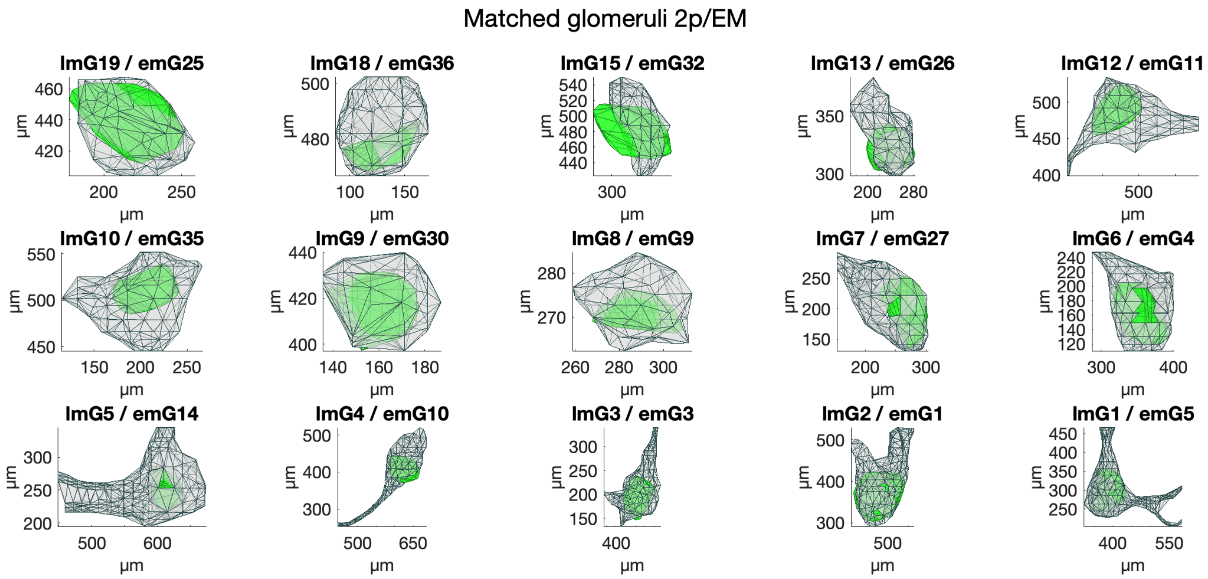

Supp. Fig. 10
Matched glomerular regions of interest between 2-photon and SBEM.
Individual plots for all matched glomerular ROI pairs 2-photon *in vivo* (green) / SBEM (grey).

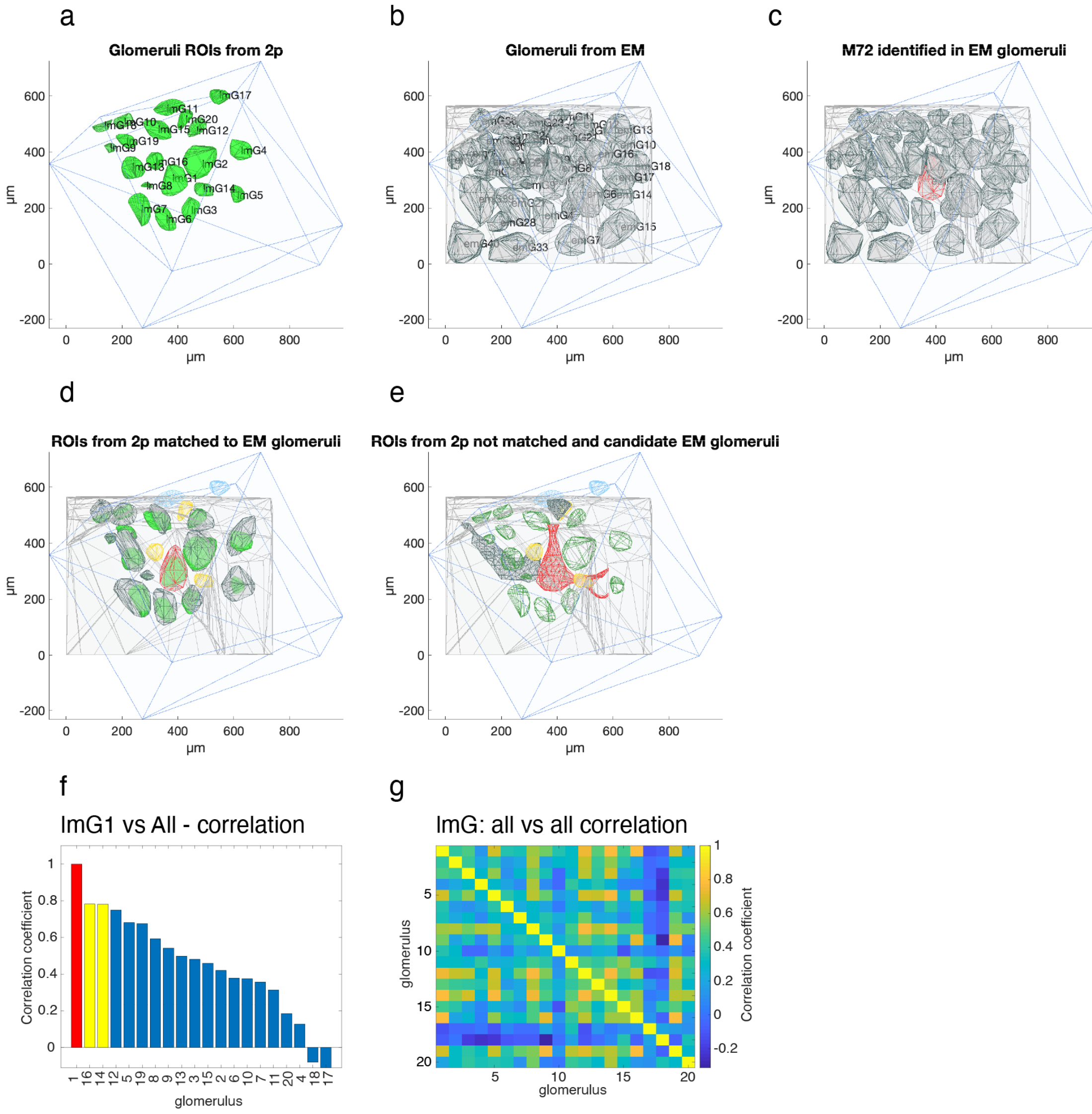

Supp. Fig. 11

Tracking glomerular regions of interest between 2-photon and SBEM

(a) Glomerular ROIs segmented in the 2-photon *in vivo* dataset. ROIs shown in green, with their respective 2-photon identifier tags overlapped. The field of view covered by the 2-photon dataset is shown in blue. **(b)** Glomerular ROIs segmented in the EM dataset. ROIs shown in grey, with their EM identifier tags overlapped. The field of view covered by the EM dataset is shown in pale grey. **(c)** The glomerulus M72 is highlighted in red. **(d)** Matched glomerular ROIs between 2-photon and EM. Only matched EM ROIs are shown, along with matched 2-photon (green), unmatched 2-photon (yellow) and 2-photon ROIs that fall outside the EM field of view (blue). **(e)** Detailed contours of EM ROI candidates for the unmatched 2-photon ROIs. Two unmatched ROIs fall very close to axon bundles associated with the M72 glomerulus, which also contains a matched ROI. **(f)** Cross-correlation between the activity profile recorded in the M72 ROI (red) and in all other ROIs. The columns of the two nearby unmatched ROIs are highlighted in yellow. **(g)** Cross-correlation of the activity profile recorded across all glomeruli.

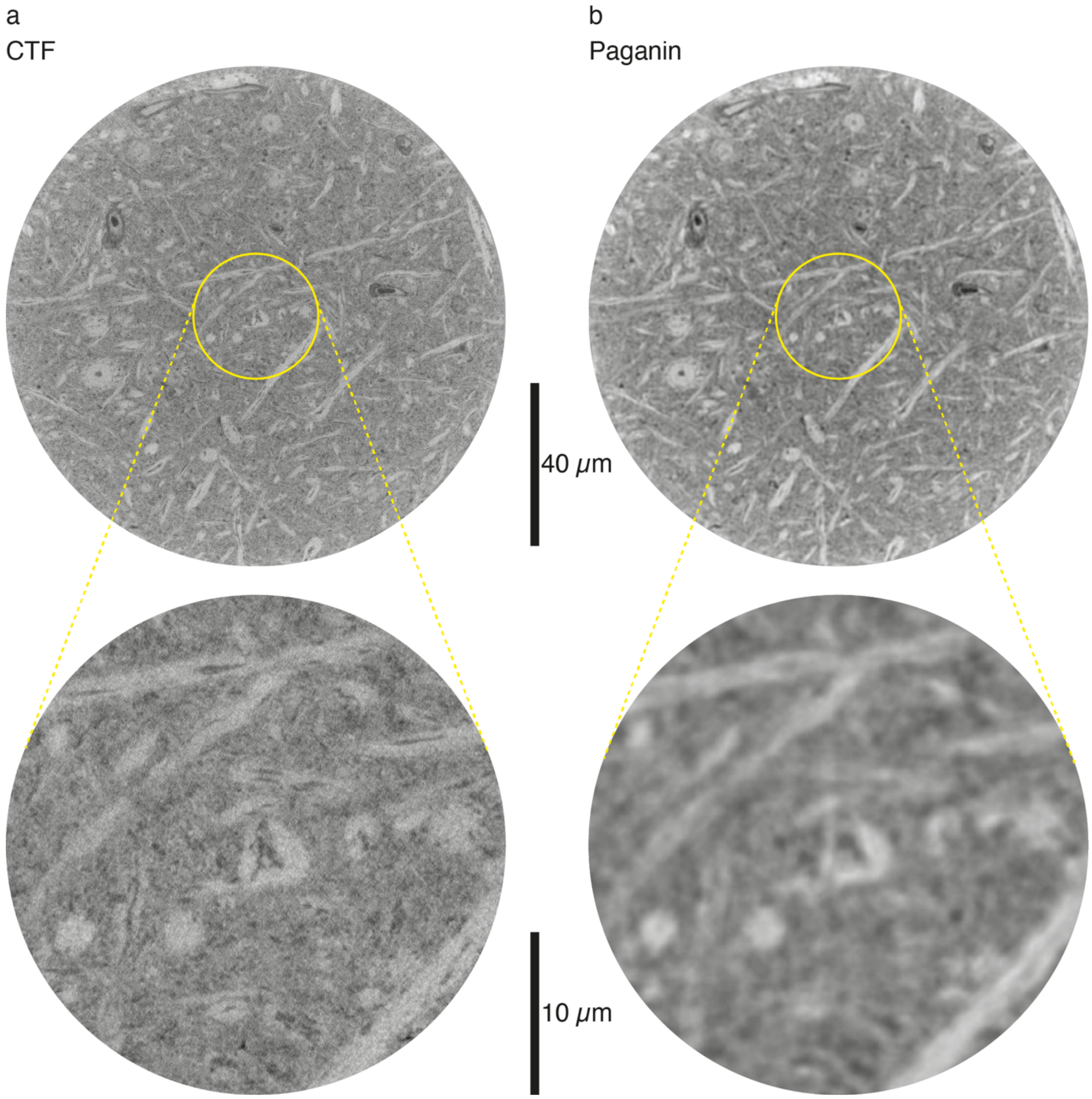

Supp. Fig. 12
Comparison of tomography reconstruction algorithms.
A specimen containing mouse olfactory bulb external plexiform layer was imaged at the nano-imaging beamline ID16A (ESRF) and reconstructed with two algorithms: CTF **(a)** and Paganin **(b)**. The same region was located in both reconstructions. The reconstructed field of view is shown in the top panels, and close-up details from each reconstruction method are shown in the bottom panels, respectively.

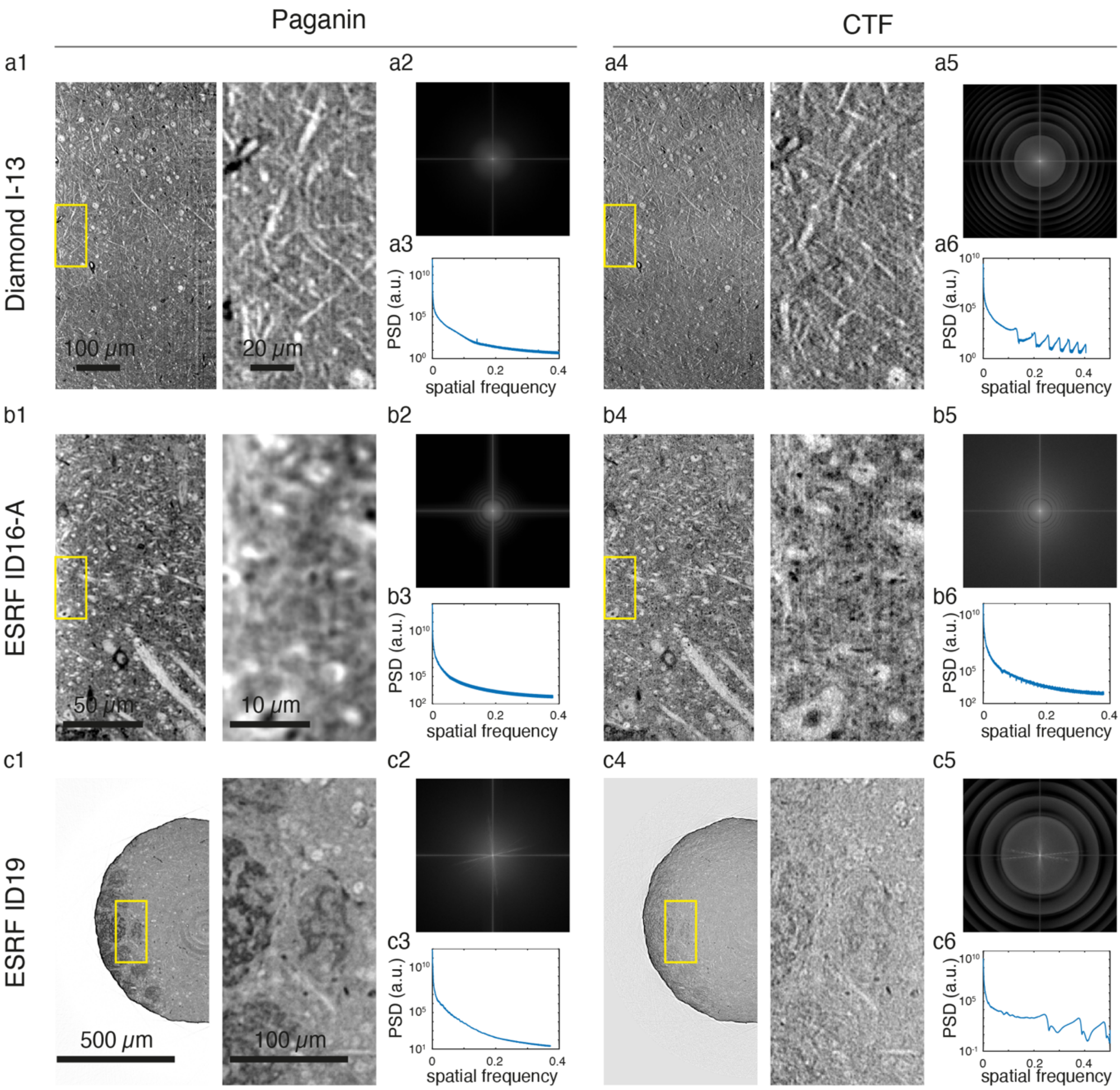

Supp. Fig. 13

Comparison of reconstructions with CTF and Paganin phase-retrieval.

(a1) Reconstructed image, **(a2)** power spectral density (PSD), and **(a3)** azimuthally-averaged PSDs after Paganin phase-retrieval for data from the microtomography beamline Diamond I13-2. **(a4-a6)** same as **(a1-a3)** but for CTF phase-retrieval.

(b) same as **(a)** but for nano-holotomography at ESRF ID16-A

(c) same as **(a)** but for the microtomography beamline ESRF ID19 for a 800 µm cylindrical sample.

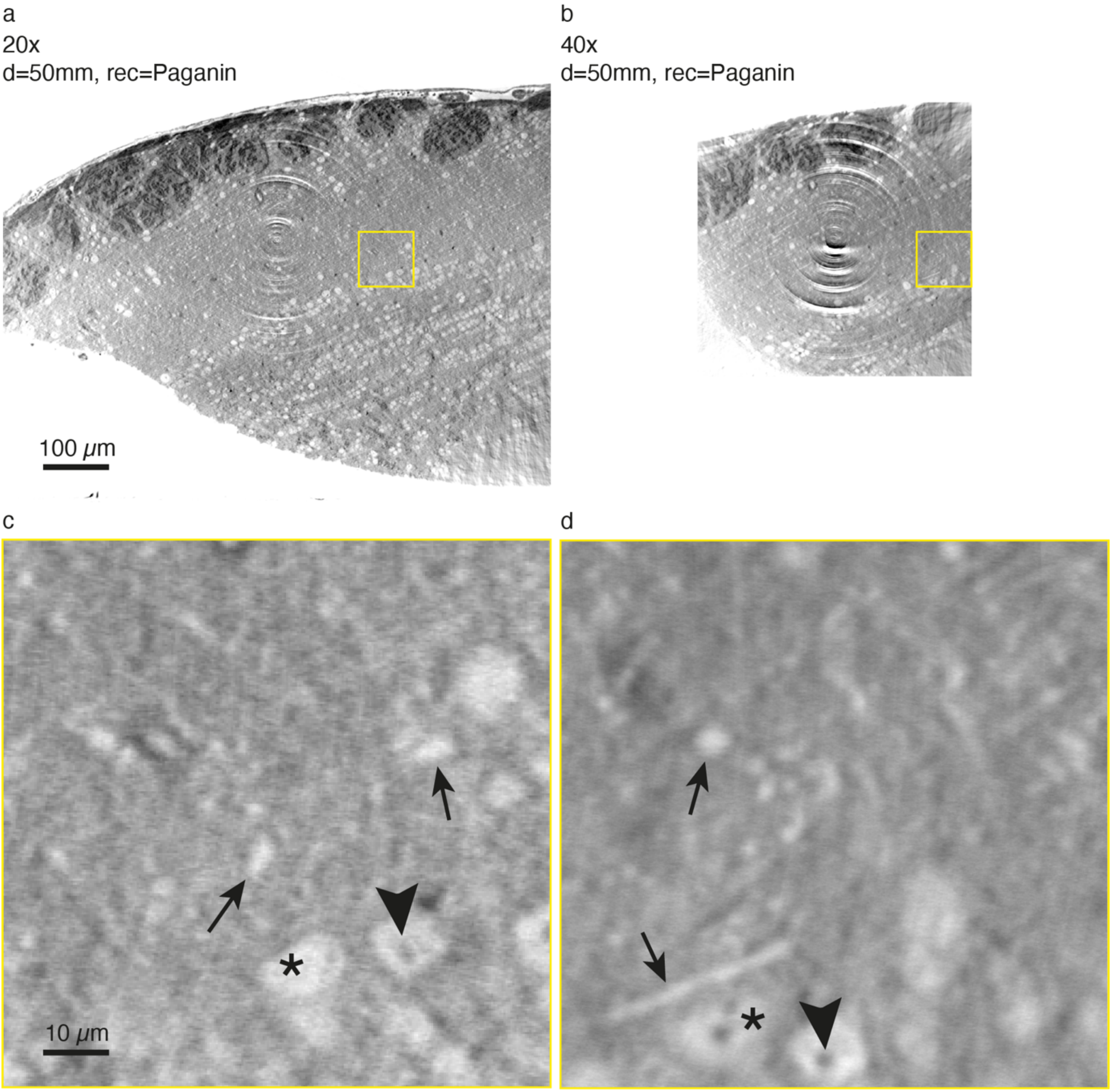

Supp Fig 14
SXRT at 20x and 40x
(a, b) SXRT datasets obtained with 20x **(a)** and 40x **(b)** objectives on the same specimen at the TOMCAT beamline. Full tile field of view is shown for each case. Both datasets were acquired with a distance of 50mm between sample and detector, and the tomograms reconstructed using Paganin filtering.
(c, d) Enlarged detail of the zones indicated in the 20x dataset **(c)** and 40x **(d)**, respectively. Cell nuclei (asterisks), nucleoli (arrowheads) and dendritic processes (arrows) are clearly defined in both, with the 40x dataset enabling improved delineation of these features.

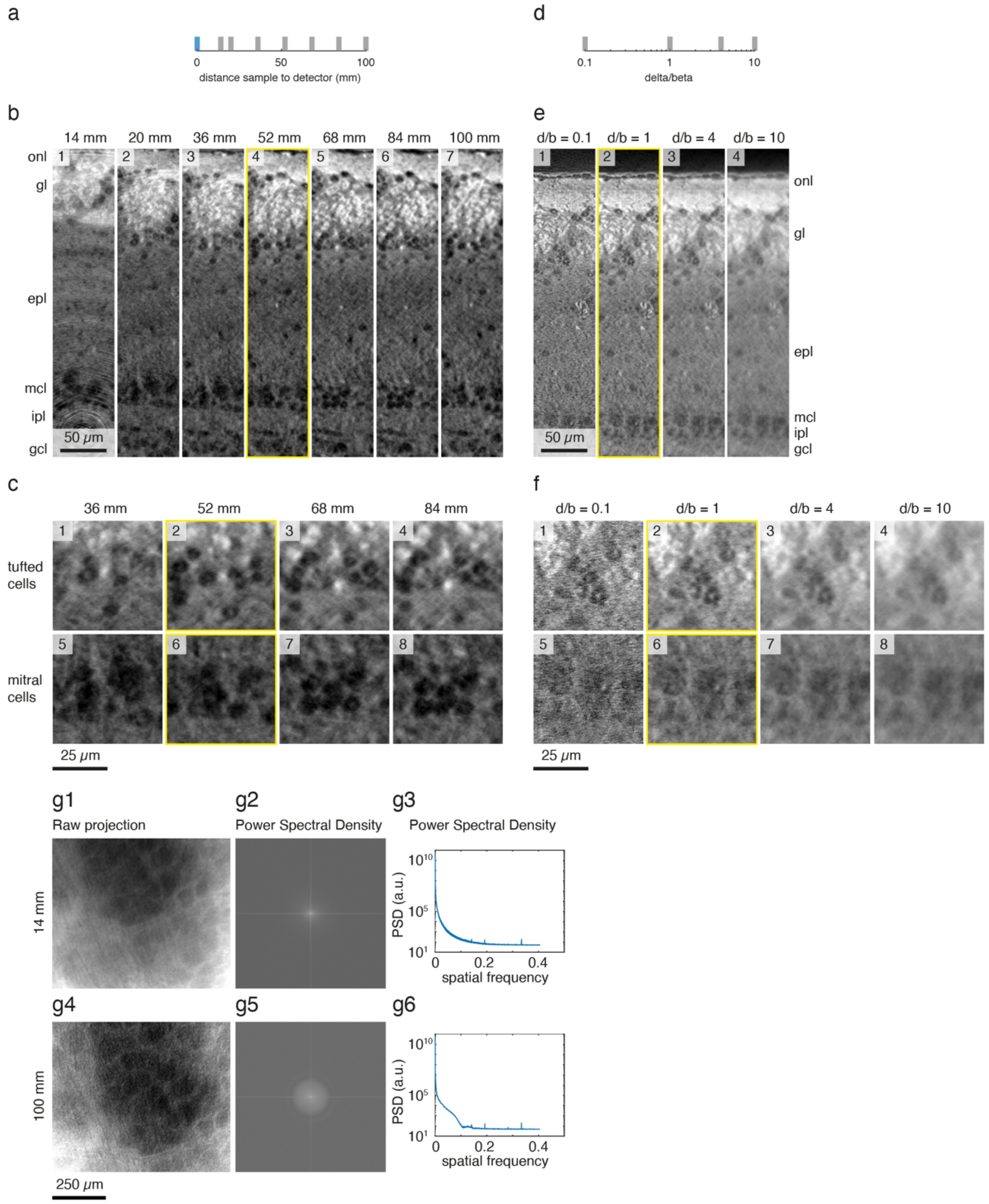

Supp. Fig. 15
Optimisation of acquisition and reconstruction parameters for SXRT datasets.
(a-c) Different sample-detector distances **(a)** were tested at the different beamlines (cases shown relate to tests performed at I13-2). Using the same sample, a tomogram was acquired at each distance, reconstructed, and the same region was located in all reconstructions. The histological patterns revealed across ~250 µm landscapes **(b)** enabled judging quality variations across extreme parameter values, but examining details at the 20 µm scale such as cell nuclei **(c)** was necessary to decide on the optimal sample-detector distance (highlighted). **(d-f)** Different delta/beta ratios **(d)** were tested empirically at the different beamlines (cases shown relate to tests performed at I13-2). A raw tomogram (acquired at the optimal sample-detector distance), was reconstructed several times using the different delta/beta ratios and the same region was located in all reconstructions. The histological patterns revealed across ~250

μm landscapes **(e)** enabled judging quality variations across extreme parameter values, but examining details at the 20 μm scale such as cell nuclei **(f)** was necessary to decide on the optimal delta/beta ratio in the implemented reconstruction algorithm (highlighted).
(g) Raw projection **(g1)** and corresponding PSD **(g2)** and azimuthally-averaged PSD **(g3)** for a sample-detector distance of 14 mm at Diamond I13-2. **(g4-g6)** same as **(g1-g3)** but for a sample-detector distance of 100 mm. Note the gain in information for larger propagation distance.
onl, olfactory nerve layer; *gl*, glomerular layer; *epl*, external plexiform layer; *mcl*, mitral cell layer; *ipl*, inner plexiform layer; *gcl*, granule cell layer.

a

b

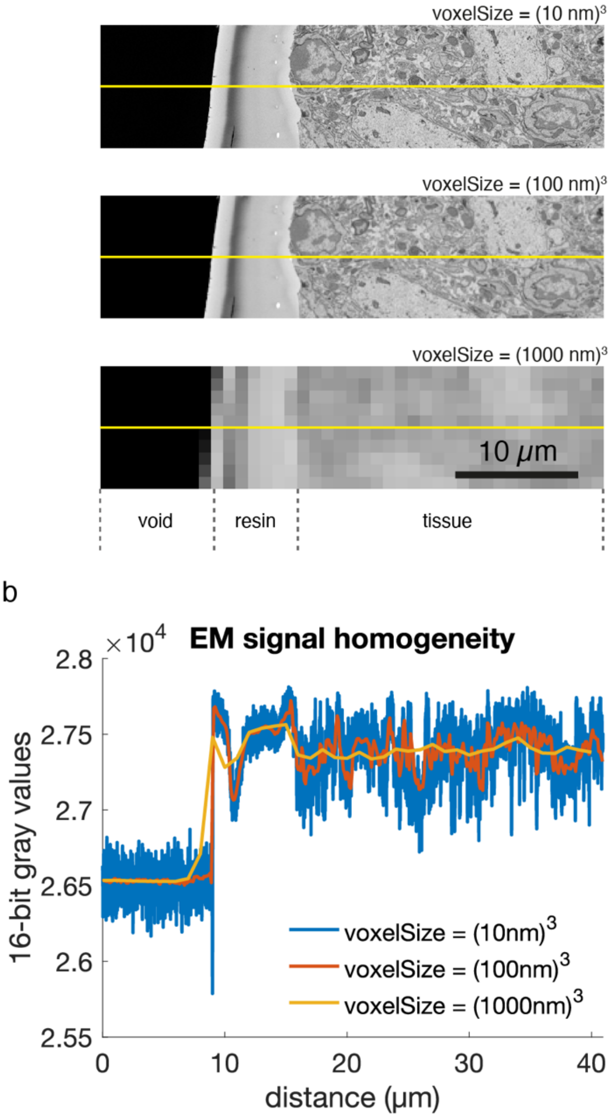

Supp. Fig. 16
Sample homogeneity.
(**a**) Backscattered electron image obtained with SBEM of the region at the edge of the blockface (stained brain tissue, mouse olfactory bulb, EPL). The field of view contains the void before the specimen begins, and the specimen composed of an external layer of resin with conductive silver followed by the stained tissue. The originally acquired image (top) was digitally downscaled 10 and 100-fold (bottom). The grey values of the horizontal midline of each image were recorded. (**b**) Signal recorded at the original and downscaled images.

Specimen	animal_ID	age (w)	gender	hemisphere	location	figures
C435	ASAG21.3a	12	male	left	first dorsal slice, olf. bulb	SuppF7, SuppF13, SuppF14, SuppF15
C417	ASAH16.9a	8.3	male	right	first dorsal slice, olf. bulb	SuppF12, SuppF13
C406	ASAH33.1a	9.3	male	left	first dorsal slice, olf. bulb	SuppF5
C410	ASAH33.1b	9.3	male	left	first dorsal slice, olf. bulb	SuppF5
C319	ASAK1.2h	13.2	male	left	first dorsal slice, olf. bulb	SuppF16
C458	ASAM11.3c	9	male	left	first dorsal slice, olf. bulb	SuppF5
C525	ASAM15.2a	10	male	left	first dorsal slice, olf. bulb	1, 2, 3, 6, 7, SuppF1, SuppF2, SuppF3, SuppF4, SuppF5, SuppF6, SuppF7, SuppF8, SuppF10, SuppF11
C414	ASAM3.2a	10	male	left	first dorsal slice, olf. bulb	SuppF5
C432	ASAM3.2g	11	female	left	first dorsal slice, olf. bulb	SuppF3, SuppF5, SuppF7
C433	ASAM3.2g	11	female	right	first dorsal slice, olf. bulb	SuppF5
C488	ASAM3.9d	8	male	left	first dorsal slice, olf. bulb	SuppF5
C450	ASAM5.4d	12	female	left	first dorsal slice, olf. bulb	SuppF5
C420	ASAM7.3i	12	female	left	first dorsal slice, olf. bulb	SuppF5
C543	ASAU7.2a	10	male	n/a	coronal slice, cortex and striatum	5, SuppF1
C555	ASAU7.2c	10	male	n/a	coronal slice, cortex and anterior hippocampus	5, SuppF1
C556	ASAU7.2c	10	male	n/a	coronal slice, cortex and medial hippocampus	4, SuppF1, SuppF3, SuppF4, SuppF9
C557	ASAU7.2c	10	male	n/a	coronal slice, cerebellum	5, SuppF1
C046	BRAC	12	male	n/a	coronal slice, olfactory bulb	SuppF6

Supplementary Table 1.
Samples
Details of all specimens reported in the study. Specimens are ordered by animal identity, and all specimens belonging to the same animal display the same background on the first column.

	Diamond-I3-2	SLS-TOMCAT	ESRF-ID19	ESRF-ID16A
**Scintillator**	GGG:Eu 34 µm	LuAG:Ce 20 µm	LuAG:Ce 25 µm	GGG:Eu 23 µm
**Camera**	pco.edge 5.5	pco.edge 5.5	pco.edge 5.5	FreLoN 16
**Exposure time (s)**	0.4	0.3	0.1	0.33
**Number of projections**	3001	2601	1800	1900
**Total rotation (°)**	180	180	180	180
**Scan duration (min)**	20	13	3	45
**Field of view (µm)**	832	832	1300	192
**Effective pixel size (nm)**	325	325	650	94
**Effective distance (mm)**	52	50	105	38.45
**Energy (keV)**	22	21	26	33.6
**Wavelength (A)**	0.56	0.59	0.477	0.369
**Fresnel number**	0.0363	0.0358	0.0844	0.0062

Supplementary Table 2.
Experimental parameters at different beamlines.
