# Supplementary material for: Functional and multiscale 3D structural investigation of brain tissue through correlative in vivo physiology, synchrotron microtomography and volume electron microscopy
Source: Nat Commun. 2022 May 25;13:2923. doi: 10.1038/s41467-022-30199-6 (PMC9132960; doi:10.1038/s41467-022-30199-6)
Supplement: Supplementary file 3 — Reporting Summary [file 41467_2022_30199_MOESM3_ESM.pdf]

nature portfolio

Corresponding author(s): Carles Bosch and Andreas T. Schaefer

Last updated by author(s): 2021/12/30

Reporting Summary

Nature Portfolio wishes to improve the reproducibility of the work that we publish. This form provides structure for consistency and transparency in reporting. For further information on Nature Portfolio policies, see our Editorial Policies and the Editorial Policy Checklist.

Statistics

For all statistical analyses, confirm that the following items are present in the figure legend, table legend, main text, or Methods section.

n/a	Confirmed	
☐	☒	The exact sample size (*n*) for each experimental group/condition, given as a discrete number and unit of measurement
☐	☒	A statement on whether measurements were taken from distinct samples or whether the same sample was measured repeatedly
☐	☒	The statistical test(s) used AND whether they are one- or two-sided<br>*Only common tests should be described solely by name; describe more complex techniques in the Methods section.*
☒	☐	A description of all covariates tested
☐	☒	A description of any assumptions or corrections, such as tests of normality and adjustment for multiple comparisons
☐	☒	A full description of the statistical parameters including central tendency (e.g. means) or other basic estimates (e.g. regression coefficient) AND variation (e.g. standard deviation) or associated estimates of uncertainty (e.g. confidence intervals)
☐	☒	For null hypothesis testing, the test statistic (e.g. F, t, r) with confidence intervals, effect sizes, degrees of freedom and P value noted<br>*Give P values as exact values whenever suitable.*
☒	☐	For Bayesian analysis, information on the choice of priors and Markov chain Monte Carlo settings
☒	☐	For hierarchical and complex designs, identification of the appropriate level for tests and full reporting of outcomes
☒	☐	Estimates of effect sizes (e.g. Cohen's *d*, Pearson's *r*), indicating how they were calculated

Our web collection on statistics for biologists contains articles on many of the points above.

Software and code

Policy information about availability of computer code

Data collection	2-photon data was acquired with commercially available Scientifica software (SciScan 1.3).<br>Epifluorescence data was acquired using the commercially available ZEISS ZEN 2011 software (v1.0.0.0).<br>LXRT tomograms were acquired in a ZEISS Versa 510 micro-CT using ZEISS Scout and Scan (v. 16.1.13038.43540) and reconstructed using ZEISS Reconstruct (v. 16.1.13038.43540).<br>SXRT tomograms were acquired using GDA (at Diamond, http://www.opengda.org/) and reconstructed with savu (at Diamond I13-2, https://github.com/DiamondLightSource/Savu) and GridRec (at PSI TOMCAT, https://github.com/esther279/Gridrec-MS). At ID19 data was collected using Bliss (https://bliss.gitlab-pages.esrf.fr/bliss/master/) and reconstructed with Nabu (https://tomotools.gitlab-pages.esrf.fr/nabu/). At ID16A data was acquired using Spec and Tango (https://www.tango-controls.org/), and reconstructed using Octave and PyHST (https://software.pan-data.eu/software/74/pyhst2). Reconstructed tomograms of the same specimen were stitched using an implementation of NRStitcher (https://github.com/arttumiettinen/pi2).<br>SBEM datasets were acquired on a variable pressure Sigma SEM 3View2 using SBEMimage software (https://github.com/SBEMimage) and on a high vacuum Merlin SEM 3View2 using ZEISS SmartSEM (version 5.07) and Gatan DigitalMicrograph (version 3.32.2403.0).<br><br>All datasets were converted into a pyramidal file format using Voxelytics and explored and annotated through webKnossos (https://github.com/scalableminds/webknossos).

Data analysis

Data analysis was performed using Python (version 3.8.3), Fiji (ImageJ 1.53c), suite2p, MATLAB (2019a) and Amira (6.7.0). The toolbox for warping skeletons as described in methods section "Warping" is based on BigWarp (https://github.com/saalfeldlab/bigwarp) and its MATLAB implementation derived from https://gitlab.mpcdf.mpg.de/connectomics/L4dense. This implementation is accessible for public use alongside the warping landmarks, datasets and annotations described in this study, in https://github.com/FrancisCrickInstitute/warpAnnotations (https://doi.org/10.5281/zenodo.6342309).

For manuscripts utilizing custom algorithms or software that are central to the research but not yet described in published literature, software must be made available to editors and reviewers. We strongly encourage code deposition in a community repository (e.g. GitHub). See the Nature Portfolio guidelines for submitting code & software for further information.

Data

Policy information about availability of data

All manuscripts must include a data availability statement. This statement should provide the following information, where applicable:

- Accession codes, unique identifiers, or web links for publicly available datasets
- A description of any restrictions on data availability
- For clinical datasets or third party data, please ensure that the statement adheres to our policy

Warping landmarks, datasets, annotations and code is accessible for public use through the repository: https://github.com/FrancisCrickInstitute/warpAnnotations (https://doi.org/10.5281/zenodo.6342309)

Field-specific reporting

Please select the one below that is the best fit for your research. If you are not sure, read the appropriate sections before making your selection.

☒ Life sciences ☐ Behavioural & social sciences ☐ Ecological, evolutionary & environmental sciences

For a reference copy of the document with all sections, see nature.com/documents/nr-reporting-summary-flat.pdf

Life sciences study design

All studies must disclose on these points even when the disclosure is negative.

Sample size

For hippocampal CA1 apical tracing analyses:
In the region studied, the CA1 expanded <150µm in the radial axis and the transverse axis was parallel to the x,y plane in the SBEM dataset. The dataset extended 40 µm in depth (antero-posterior axis), in order to fully contain cell somata and their apical projections. Since distinct neuronal populations distribute along the transversal axis (e.g. CA1a vs CA1c), we aimed to constrain the analysis to only one subregion of CA1. Accordingly, we defined a region of interest marginally larger in the transverse axis than the radial thickness of the CA1: All CA1 somata contained along 200µm in the transverse axis and within 40 µm in the antero-posterior axis of the pyramidal cell layer were initially seeded in the ground truth low-resolution EM dataset (n0=175 somata). Apical dendrites could be identified in n=90 of these somata in both EM and SXRT datasets and that group conformed the sample size for analysing the accuracy in tracing apical dendrites in SXRT. We considered this sample size sufficient to capture the variability of morphologies of apical dendrites of CA1 pyramidal neurons since this sample contained >50% of all neurons in that region.

For hippocampal CA1 spine density analyses:
The stratum radiatum region imaged at high resolution was designed to contain the apical dendrites of ~10 SXRT reconstructed neurons. The trimmed blockface, with the apical dendrites evolving along the x axis, allowed targeting a field of view perpendicular to the trajectory of CA1 apical dendrites. To avoid any local heterogeneities due to soma positions in the pyramidal layer, the dataset had to cover the apical dendrite field of several adjacent soma widths. A 80µm-wide dataset was deemed sufficient in that regard, while 40µm in z should contain the dendrite of z-centered somata through the entire stratum radiatum.
The centering of the above-mentioned CA1 bounding box was chosen so its dendrites would exceedingly cover the width (80 µm) of the field of view of the high-resolution EM dataset. All seeded CA1 pyramidal neurons whose apical dendrite could be traced in SXRT until inside that high-resolution SBEM volume were selected for spine analysis (n=7). The depth (position in the radial axis) of the soma of those n=7 cells covered the most populated 50% of the range of observed soma depths. Therefore, we considered that our sample should capture any effect of soma depth on the density of spines and spines with spine apparati in their dendrites.

For mitral cell apical dendrite tracing analyses:
Mitral cells are projection neurons with a prominent apical dendrite, whose somata are located in a monolayer. Mitral cells are one major type of projection neurons of olfactory bulb glomerular columns, a circuit that modularly repeats its structure across the olfactory bulb. In a previous study of one genetically-identified glomerular column, we determined that the circuit contained on average 9 mitral cells whose somata scattered across (0.5 mm)^2 laterally in the mitral cell layer (Schwartz et al. 2018). For this current study, we assumed that the heterogeneity of morphologies of their apical dendrites could therefore be captured by a randomly selected group of 50 mitral cells in the dorsal olfactory bulb. Finally, the observed variability in apical dendrite traceability in SXRT of CA1 pyramidal neurons supported this experimental design.

For glomerular region of interest deambiguation
A typical experimental setup aiming to report the physiological activity of neurons involved in neighbouring glomerular columns at a temporal resolution of 2.5Hz covers a tissue volume of (464µm)^2 x 440µm in z, sampling that z-volume in 12 planes, which involves imaging the

glomerular layer reliably in 1 plane. The experiment reported identified n_2P=20 glomerular regions of interest based solely on 2P data. We considered that the share of glomeruli matched across modalities for that one experiment would be a good indicator of the expected yield of similar correlative multimodal experiments.

for resolution measurements
In all cases, cube regions of interest were obtained as large as possible so features deemed to be resolved would be fully contained in reasonable numbers inside the region of interest and at least 3 replicates could be sampled within the same specimen. As a result, those regions expanded (500 voxels)^3 for all modalities except in the smaller dataset LXRT, in which they expanded (100 voxels)^3. We considered that 3 comparable regions within the same dataset would represent variabilities in staining and in histology, and that therefore the sample size would be sufficient to report the resolving capacity of each technique. For SXRT, inter-specimen and inter-beamline variability was also explored (n = 2 specimens, n = 2 beamlines). We considered that this sample size would be sufficient to report large effects of either variable, if present.

for sample homogeneity measurements
To analyse recorded intensity with BSED on stained brain tissue we sampled one specimen blockface so in 4096 * 10nm pixels horizontally we could record the baseline signal for 3 sample zones: null signal, resin and tissue. That image would next be downscaled to match pixel sizes of 100 nm and 1 µm, and in each case biological features of interest should be resolved within each section. The coarser detectable biological features would be apical dendrites of 5 µm in thickness, which would be represented by 500 pixels in the original image. Therefore, the sample region was chosen so at least twice that distance (covering 1000 pixels) would be present in each sample zone, and so all regions would be traversed in the horizontal axis (direction of scanning).

Data exclusions

For hippocampal CA1 apical tracing analyses:
From the 175 seeded CA1 somata, in 132 their apicals could be traced in EM. Those somata were warped into the SXRT dataset. From these, 90 could be traced in SXRT and were all included in the analysis.

For hippocampal CA1 spine density analyses:
All cells whose apical dendrite could be traced in SXRT inside the high-resolution EM were included in the analysis (n=7 cells). Their dendrites and spines were fully annotated and the dendrites classified as trunk or apical oblique. When measuring spine distributions, dendritic segments containing <30 spines were excluded from analysis to ensure a large enough sample size from any extracted measurement.

For mitral cell apical dendrite tracing analyses:
All mitral cell somata contained in the SBEM dataset were seeded (n0 = 372). n = 50 of these were chosen randomly and traced in both EM and SXRT. All 50 cells could be traced in EM and were all included in the analysis. These same 50 cells were submitted for tracing in the SXRT dataset to 3 independent tracers. On average, each tracer provided annotations for n' = 44 cells. All these cells were included in the analysis.

For glomerular region of interest deambiguation
All glomeruli in the dataset were identified in the distinct imaging modalities alone (n_2p = 20, n_EM = 41). All annotations were included in the analysis.

for resolution measurements
Regions of interest of the defined sizes were designed in each dataset. The data in all sampled regions was assessed to confirm all regions fully contained tissue before running the analysis. All regions were included in the analysis.

for sample homogeneity measurements
All pixels in the midline were included in the analysis.

Replication

For hippocampal CA1 apical tracing analyses:
A second dataset of hippocampal tissue was also acquired and apical dendrites of CA1 pyramidal neurons were visually traceable as well.

For hippocampal CA1 spine density analyses:
The reported experiment is the only replicate we performed.

For mitral cell apical dendrite tracing analyses:
We acquired multiple similar samples with SXRT at distinct synchrotron beamlines, and apical dendrites of mitral cells were always evident. We provide in supplementary material n = 10 independent samples from n' = 9 different mice in which apical dendrites could be followed.

For glomerular region of interest deambiguation
The reproducibility of the correlative multimodal imaging pipeline was performed multiple times. We report in supplementary n = 10 replicates arising from n = 9 mice for which all cases the genetically-targeted glomerulus could be identified in SBEM.

for resolution measurements
We report all replicates performed to date.

for sample homogeneity measurements
The pattern reported has been widely replicated in conventional acquisition sessions with multiple samples and microscopes.

Randomization

For hippocampal CA1 apical tracing analyses:
All cells in the defined bounding box were traced. Tracing performance was assumed independent of cell position and tracing order. All cells were traced by the same tracer in both SXRT and EM datasets. Experimental groups were solely determined by the imaging modality of the dataset in which the features were segmented (SXRT, SBEM).

For hippocampal CA1 spine density analyses:
Dendrites were classified as trunk or apical oblique according to the following criteria: trunk dendrites display thick diameters (>=2µm) and trajectories consistently perpendicular to the pyramidal layer, and branch no more than once defining an accute branching angle of ~60° between the two final branches. Apical oblique dendrites arise perpendicular to the trajectory of the trunk dendrite within the stratum radiatum, have thinner diameters (~1µm), can branch multiple times and resulting in asymmetrical branches.

All annotated spines containing a spine apparatus were given that additional category.

For mitral cell apical dendrite tracing analyses:
Experimental groups were solely determined by the imaging modality of the dataset in which the features were segmented (SXRT, SBEM) or by the beamline in which the SXRT dataset was recorded (I13-2, TOMCAT).

For glomerular region of interest deambiguation
Experimental groups were solely determined by the imaging modality of the dataset in which the features were segmented (2P, SBEM).

for resolution measurements
Experimental groups were solely determined by the imaging modality of the dataset in which the features were segmented (LXRT, SXRT, SBEM).

for sample homogeneity measurements.
Regions were defined based on the presence of tissue or resin (resulting in void, resin, tissue). All pixels in each region were included in the analysis.

Blinding

For hippocampal CA1 apical tracing analyses:
Each annotation was proof-read by the tracer until satisfied. Doubtful locations were commented with a second expert tracer and resolved by consensus.
Blinding was not possible since the image modality is evident from the images to be traced themselves. It was not necessary to apply blinding since tracings were paired to their own ground truth through a correlative experiment.

For hippocampal CA1 spine density analyses:
The tracer was blind to cell depth when annotating dendrites and dendritic spines.

For mitral cell apical dendrite tracing analyses:
Blinding was not possible since the image modality is evident from the images to be traced themselves. It was not necessary to apply blinding since tracings were paired to their own ground truth through a correlative experiment. Each annotation was proof-read by the tracer until satisfied. For doubtful locations in annotating apical dendrites in SBEM, consensus was obtained by majority vote across 3 tracers.

For glomerular region of interest deambiguation
Blinding was not possible since the image modality is evident from the images to be traced themselves. Tracers were blind to totals of glomeruli identified in distinct modalities (2P, SBEM).

for resolution measurements
Blinding was not possible since the image modality is evident from the images to be traced themselves. All histological regions were densely sampled in large regions of interest, ensuring that the resolution metrics obtained would be robustly representative of the biological features aimed to be resolved by each dataset and modality.

for sample homogeneity measurements
Blinding was not possible since the sample zones were evident. Blinding was not necessary since the result could be readily reproduced in multiple equivalent regions.

Reporting for specific materials, systems and methods

We require information from authors about some types of materials, experimental systems and methods used in many studies. Here, indicate whether each material, system or method listed is relevant to your study. If you are not sure if a list item applies to your research, read the appropriate section before selecting a response.

Materials & experimental systems

n/a	Involved in the study
☒	☐ Antibodies
☒	☐ Eukaryotic cell lines
☒	☐ Palaeontology and archaeology
☐	☒ Animals and other organisms
☒	☐ Human research participants
☒	☐ Clinical data
☒	☐ Dual use research of concern

Methods

n/a	Involved in the study
☒	☐ ChIP-seq
☒	☐ Flow cytometry
☒	☐ MRI-based neuroimaging

Animals and other organisms

Policy information about studies involving animals; ARRIVE guidelines recommended for reporting animal research

Laboratory animals

Animals in this study were all 8-13 weeks-old mice. For experiment C525, we used a male transgenic mouse resulting of MOR174/9-eGFP crossed into M72-IRES-ChR2-YFP (JAX stock #021206) crossed into a Tbet-cre driver line (JAX stock #024507) crossed with a GCaMP6f reporter line (JAX stock #028865). For experiment C556, we used a male mouse of C57Bl/6 background. For the rest of the experiments, we used mice of either gender of C57Bl/6 background. All animal protocols were approved by the Ethics Committee of the board of the Francis Crick Institute and the United Kingdom Home Office under the Animals (Scientific Procedures) Act 1986.

Wild animals	No wild animals were used in this study.
Field-collected samples	No field-collected samples were used in this study.
Ethics oversight	All animal protocols were approved by the Ethics Committee of the board of the Francis Crick Institute and the United Kingdom Home Office under the Animals (Scientific Procedures) Act 1986.

Note that full information on the approval of the study protocol must also be provided in the manuscript.
